# Factors affecting serum concentration of vancomycin in critically ill oliguric pediatric patients receiving continuous venovenous hemodiafiltration

**DOI:** 10.1371/journal.pone.0199158

**Published:** 2018-06-21

**Authors:** Bongjin Lee, Soo Jung Kim, June Dong Park, Jiun Park, Ae Hee Jung, Sun Hoi Jung, Yu Hyeon Choi, Hee Gyung Kang, Il Soo Ha, Hae Il Cheong

**Affiliations:** 1 Department of Pediatrics, Seoul National University College of Medicine, Seoul, Republic of Korea; 2 Department of Pediatrics, Chungnam National University Hospital, Daejeon, Republic of Korea; 3 Department of Pharmacy, Seoul National University Hospital, Seoul, Republic of Korea; Hospital Universitario de la Princesa, SPAIN

## Abstract

Vancomycin is known to be unintentionally eliminated by continuous renal replacement therapy, and the protein bound fraction of vancomycin is also known to be different in adults and children. However, there are only a few studies investigating the relationship between the dose of continuous venovenous hemodiafiltration (CVVHDF) parameters and serum concentration of vancomycin in pediatric patients. The aim of this study was to determine clinical and demographic parameters that significantly affect serum vancomycin concentrations. This retrospective cohort study was conducted at a pediatric intensive care unit in a tertiary university children’s hospital. Data from oliguric patients who underwent CVVHDF and vancomycin therapeutic drug monitoring were collected. The correlation between factors affecting serum concentration of vancomycin was analyzed using mixed effect model. A total of 177 serum samples undergoing vancomycin therapeutic drug monitoring were analyzed. The median age of study participants was 2.23 (interquartile range, 0.3–11.84) years, and 126 (71.19%) were male patients. Serum concentration of vancomycin decreased significantly as the effluent flow rate (EFR; *P* < 0.001), dialysate flow rate (DFR; *P* = 0.009), replacement fluid flow rate (RFFR; *P* = 0.008), the proportion of RFFR in the sum of DFR and RFFR (*P* = 0.025), and residual urine output increased. The adjusted R^2^ of the multivariate regression model was 0.874 (P < 0.001) and the equation was as follows: Vancomycin trough level (mg/L) = (0.283 × daily dose of vancomycin [mg/kg/d]) + (365.139 / EFR [mL/h/kg])–(15.842 × residual urine output [mL/h/kg]). This study demonstrated that the serum concentration of vancomycin was associated with EFR, DFR, RFFR, the proportion of RFFR, and residual urine output in oliguric pediatric patients receiving CVVHDF.

## Introduction

Vancomycin is the first-choice drug for methicillin resistant *Staphylococcus aureus* infections, and is one of the most frequently used antibiotics in the intensive care unit (ICU) [1, 2]. Since vancomycin is excreted not only through the kidneys but also through continuous renal replacement therapy (CRRT), studies have reported that the serum concentration of vancomycin is affected in patients receiving CRRT [3–7]. In addition, serum vancomycin concentrations are known to be affected by the dose of CRRT [8].

Maintaining an adequate level of antibiotic in patients with infectious diseases is undoubtedly important. Failure to maintain adequate serum levels results in treatment failure or drug toxicity, and for critically ill patients, the outcome may be fatal [6, 7, 9]. Considering that most patients that are administered with vancomycin whilst receiving CRRT are critically ill, maintaining therapeutic serum concentrations of vancomycin is one of the most important factors related to successful treatment outcome in these patients. For this reason, several studies have been conducted on the pharmacokinetics and the optimal dose of vancomycin during the application of CRRT [6, 10–15]. However, most studies have focused on the effects of either hemofiltration or hemodialysis [10, 13]; and there are relatively few studies focused on continuous venovenous hemodiafiltration (CVVHDF) involving the interaction of continuous venovenous hemofiltration (CVVH) and continuous venovenous hemodialysis (CVVHD) [6, 11].

A previous study reported that vancomycin clearance was considerably enhanced with hemofiltration compared to hemodialysis [16]. In contrast, another study reported that the difference in vancomycin clearance was insignificant between hemofiltration and hemodialysis [17]. Another study recommended higher vancomycin doses in patients receiving CVVHD than CVVH, suggesting that vancomycin clearance by hemofiltration was lower than by hemodialysis [12, 15]. Regardless, all of these studies were conducted in adults. Since the protein bound fraction of vancomycin has been reported to be lower in children than in adults [18, 19], there is a limit to applying the results from adult studies directly to children.

Therefore, this study aimed to analyze the effect of the dose of each CVVHDF parameter and clinical characteristics on the serum concentration of vancomycin in pediatric patients receiving CVVHDF.

## Materials and methods

### Study population

This retrospective cohort study was conducted at a pediatric ICU (PICU) with 24 beds, in a tertiary university children’s hospital. Patients ≤ 18 years old who received CVVHDF and were subjected to vancomycin therapeutic drug monitoring (TDM) at the PICU between January 1, 2005 and August 31, 2015 were included. Samples of vancomycin TDM which the steady-state level of vancomycin was not reached during CVVHDF application were excluded from the study; the steady state was expected to be reached after four or more doses of vancomycin were administered at equal intervals, in accordance with the Infectious Diseases Society of America guideline [20]. Only samples collected for TDM 30 minutes before the administration of vancomycin were included to obtain consistent results [20, 21]. Because extracorporeal membrane oxygenation (ECMO) has significant effects on the hemodynamics of patients and can change the serum concentration of vancomycin, patients receiving ECMO were excluded. Patients without oliguria (urine output ≥ 0.5 mL/kg/h) were also excluded because residual renal function in these patients may enhance the clearance of vancomycin [22].

### Data collection and definition

Data on patients’ age, sex, serum concentration of vancomycin, daily dose and dosing interval of vancomycin, blood flow rate (BFR), dialysate flow rate (DFR), patient fluid removal rate (PFRR), replacement fluid flow rate (RFFR), vital signs, fluid input/output balance, residual urine output, and laboratory test results were collected from the electronic medical records. All CVVHDF parameters within 24 hours prior to TDM, and if there were more than 2 values for each of the parameters, their mean value was adopted. Effluent flow rate (EFR) was defined as the sum of DFR, RFFR and PFRR; and the proportion of RFFR was defined as the proportion of RFFR in the sum of DFR and RFFR.

Prisma® or Prismaflex® Systems (Gambro, Lakewood, Colorado, USA) were used for CVVHDF, and polyacrylonitrile (AN69) membrane was used as the hemofilter. Serum concentration of vancomycin was measured using Architect iVancomycin Reagent Kit® (Abbott Laboratories, Chicago, Illinois, USA).

### Statistical analysis

The correlation between each factor and the serum concentration of vancomycin was analyzed using univariate mixed effect model regression analysis, with patients as random variables and serum concentration of vancomycin as fixed variable. Multivariate regression analysis was performed on the factors that showed significant results in the univariate regression analyses, and the model was determined by backward selection method. In the multivariate regression model, adjustment of the statistical significance was made using Bonferroni correction, and the overall type I error rate was 0.05. All statistical analyses were performed using R version 3.4.3 (R Foundation for statistical computing, Vienna, Austria).

### Ethics statement

The Seoul National University Hospital Institutional Review Board approved the study protocol (H-1508-138-697) and waived the need for written informed consent.

## Results

### Baseline characteristics of patients

**Fig 1:**
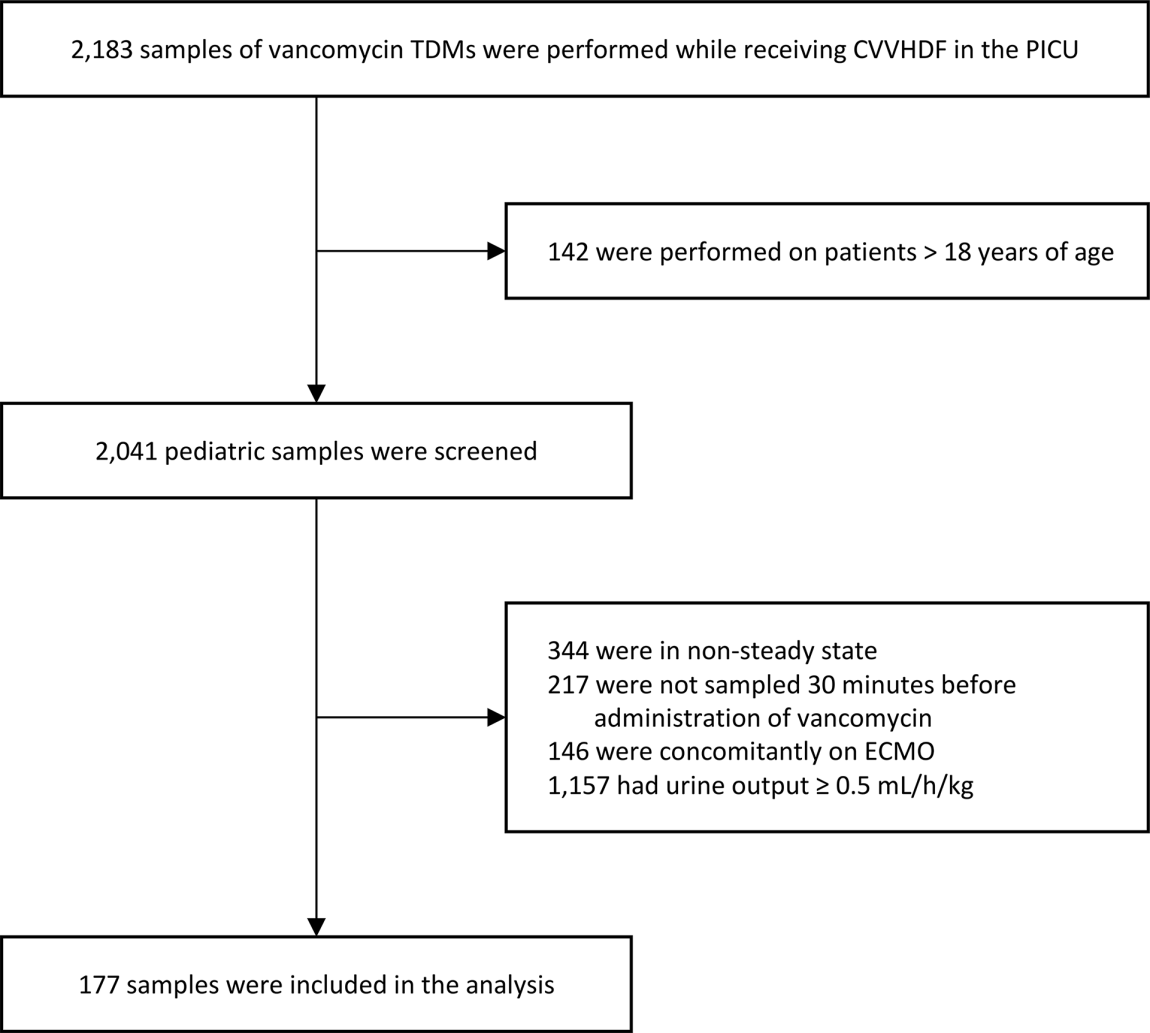
Flow chart of the study population TDM, therapeutic drug monitoring; CVVHDF, continuous venovenous hemodiafiltration; PICU, pediatric intensive care unit; ECMO, extracorporeal membrane oxygenation.

Patients who underwent vancomycin TDM while receiving CVVHDF at the PICU during the study period were screened. A total of 177 samples of vancomycin TDM from 75 patients were included in the final analyses based on the inclusion and exclusion criteria (Fig 1). The median age was 2.23 (interquartile range, 0.3–11.84) years old, and 126 (71.19%) samples were obtained from male patients. Table 1 shows the baseline characteristics of the samples included.

**Table 1:** Baseline characteristics and the relationship between each characteristic and the trough level of vancomycin.

Characteristics		No. of samples (N = 177)	Estimate	SE	*P*
Age (years)		2.23 (0.3–11.84)	-0.254	0.259	0.33
Sex	Female	51 (28.81)	Reference		
	Male	126 (71.19)	3.73	3.337	0.267
Height (cm)		73.7 (57–128.8)	-0.042	0.036	0.248
Weight (kg)		12.3 (4.9–27.8)	-0.015	0.088	0.868
Body surface area (m^2^)		0.5 (0.28–0.97)	-1.793	3.35	0.594
Underlying disease	Cardiovascular disease	58 (32.77)	Reference		
	Gastrointestinal disease	8 (4.52)	-1.244	7.746	0.873
	Genitourinary disease	22 (12.43)	0.173	5.592	0.975
	Hemato-oncologic disease	35 (19.77)	6.165	4.718	0.194
	Immunologic disease	13 (7.34)	-1.061	6.965	0.879
	Infectious disease	12 (6.78)	3.852	5.979	0.521
	Neurologic disease	3 (1.69)	-3.457	8.981	0.702
	Respiratory disease	24 (13.56)	-2.632	4.912	0.594
	Others^a^	2 (1.13)	3.25	7.953	0.684
Administration of vancomycin	Daily dose of vancomycin (mg/kg/d)	20.11 (15.42–29.81)	0.185	0.041	<0.001
	Dosing interval of vancomycin (h)	12 (12–12)	-0.443	0.089	<0.001
	Trough level of vancomycin (mg/L)	12.3 (9.4–16.6)	NA	NA	NA
CVVHDF dose	BFR (mL/min/kg)	4.13 (3.22–6.88)	-0.373	0.214	0.085
	DFR (mL/h/kg)	26.99 (23.58–34.88)	-0.175	0.065	0.009
	PFRR (mL/h/kg)	4.73 (3.29–6.5)	-0.22	0.229	0.339
	RFFR (mL/h/kg)	23.81 (12.73–39.13)	-0.089	0.033	0.008
	EFR (mL/h/kg)	57.05 (46.26–72.73)	-0.097	0.027	<0.001
	Proportion of RFFR^b^	0.45 (0.29–0.57)	-6.102	2.668	0.025
Vital signs and clinical findings	Systolic blood pressure (mmHg)	98 (93–99)	-0.07	0.049	0.154
	Diastolic blood pressure (mmHg)	52 (45–60)	-0.053	0.048	0.271
	Heart rate (beat/min)	128 (114–142)	0.021	0.041	0.614
	Respiratory rate (breath/min)	39 (30–47)	-0.134	0.077	0.085
	Body temperature (°C)	36.45 (36.3–36.97)	-0.144	1.366	0.916
	Fluid input-output balance (mL/kg)	12.11 (-19.33 to 36.19)	0.002	0.008	0.854
	Residual urine output (mL/h/kg)	0.03 (0–0.12)	-16.237	6.355	0.012
Laboratory findings	Leukocyte (×10^3^/mm^3^)	11.83 (7.24–18.19)	0.024	0.055	0.669
	Total protein (g/dL)	6.3 (5.5–7.2)	-0.081	0.614	0.895
	Albumin (g/dL)	3.56 (3.18–4.29)	0.649	0.948	0.495
	C-reactive protein (mg/dL)	5.32 (2.33–13.94)	0.061	0.101	0.545
	Procalcitonin (ng/mL)	2.4 (1.73–6.69)	0.08	0.098	0.422

Continuous data are presented as median (interquartile range), and categorical data as n (%).

SE, standard error; NA, not applicable; CVVHDF, continuous venovenous hemodiafiltration; BFR, blood flow rate; DFR, dialysate flow rate; PFRR, patient fluid removal rate; RFFR, replacement fluid flow rate; EFR, effluent flow rate

^a^Drug intoxication and skin necrosis due to extravasation of intravenous drug were included in this disease category.

^b^Proportion of RFFR in DFR + RFFR

### Factors affecting serum concentration of vancomycin

**Fig 2:**
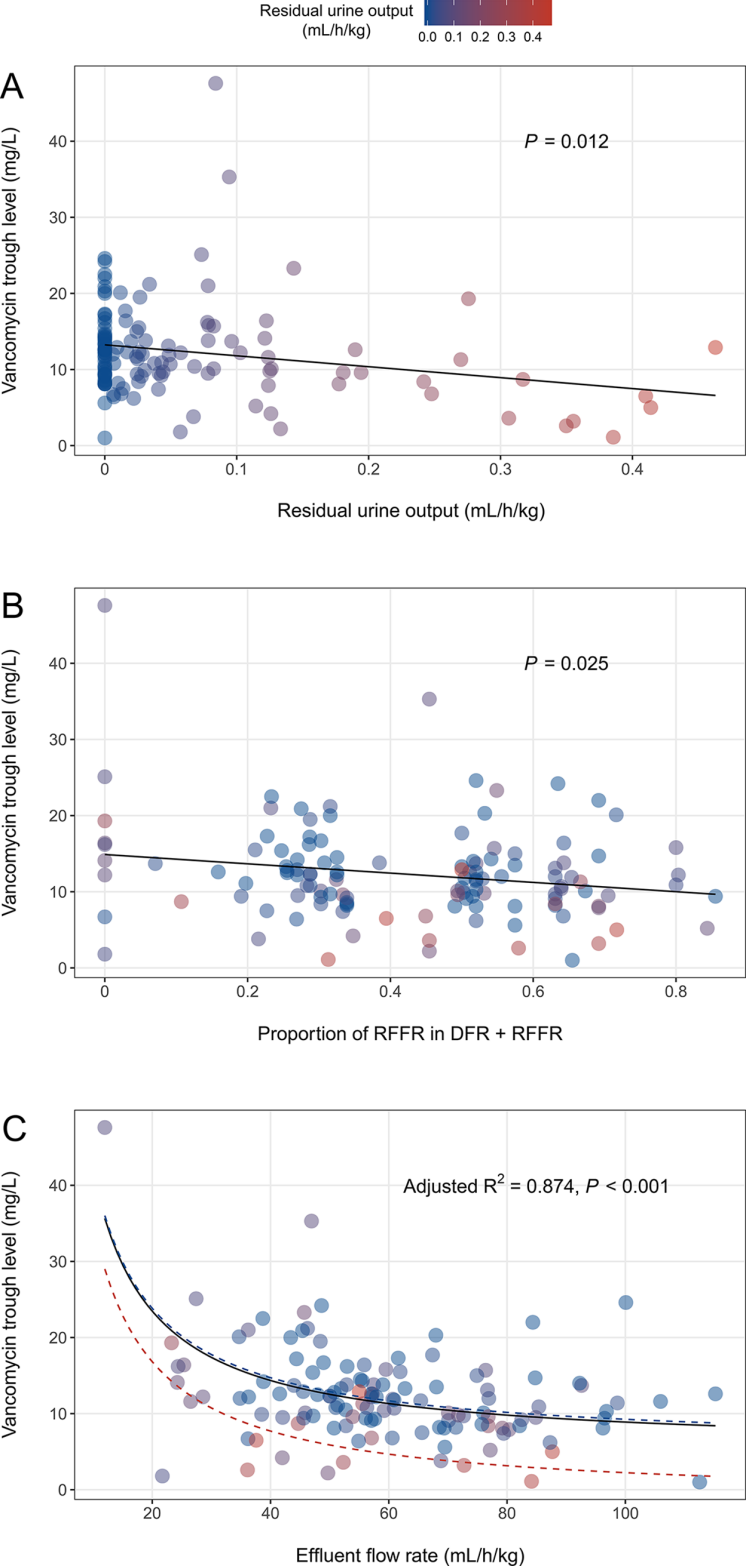
Scatterplots of the vancomycin trough level and its associated factors. The color of the spots indicates residual urine output (mL/h/kg). When the residual urine output is 0, it is blue, and when it is closer to 0.5, it is red. (A) Scatterplot of the residual urine output and vancomycin trough level showing a regression line (y = -16.24x + 17.60). (B) The line represents a linear regression line between proportion of RFFR and vancomycin trough level, with the formula ‘y = -6.102x + 14.889’. (C) The curves obtained by substituting the median value (20.11 mg/kg/d) for ‘daily dose of vancomycin’ in a multivariate regression model (Vancomycin trough level [mg/L] = 0.28 × daily dose of vancomycin [mg/kg/d] + 365.14 / effluent flow rate [mL/h/kg]– 15.84 × residual urine output [mL/h/kg]). The black solid curve is obtained by inputting 0.03 (mL/h/kg) as the median value in the residual urine output. The minimum value of 0 (mL/h/kg) is shown as a blue dashed curve, and the maximum value of 0.46 (mL/h/kg) is shown as the red dashed curve.

Univariate analyses showed that daily dose and dosing interval of vancomycin, DFR, RFFR, EFR, the proportion of RFFR, and residual urine output were significantly related to serum concentration of vancomycin. As the daily dose of vancomycin (*P* < 0.001) increased, and dosing interval of vancomycin (*P* < 0.001), DFR (*P* = 0.009), RFFR (*P* = 0.008), EFR (*P* < 0.001), the proportion of RFFR (*P* = 0.025), and residual urine output (*P* = 0.012) decreased, the serum concentration of vancomycin increased (Table 1, Fig 2A and 2B).

Multivariate analysis was performed on the significant variables found in the univariate analysis. The multivariate model derived from backward selection had four predictor variables including daily dose of vancomycin (*P* < 0.001), dosing interval of vancomycin (*P* < 0.001), EFR (*P* = 0.019), and residual urine output (*P* < 0.001). However, the *P*-value of dosing interval of vancomycin was greater than the corrected *P*-value 0.0125 through the Bonferroni method, and the final model was as follows (adjusted R^2^ = 0.874, *P* < 0.001) (Table 2, Fig 2C):

Vancomycin trough level (mg/L) = (0.283 × daily dose of vancomycin [mg/kg/d]) + (365.139 / EFR [mL/h/kg])–(15.842 × residual urine output [mL/h/kg]).

**Table 2:** Relationship between each characteristic and the trough level of vancomycin (multivariate mixed effect model regression analysis).

Characteristics	Estimate	SE	*P*
Daily dose of vancomycin (mg/kg/d)	0.283	0.035	<0.001
EFR (mL/h/kg)	365.139	38.655	<0.001
Residual urine output (mL/h/kg)	-15.842	4.312	<0.001

SE, standard error; EFR, effluent flow rate

### Vancomycin trough level group comparison

**Fig 3:**
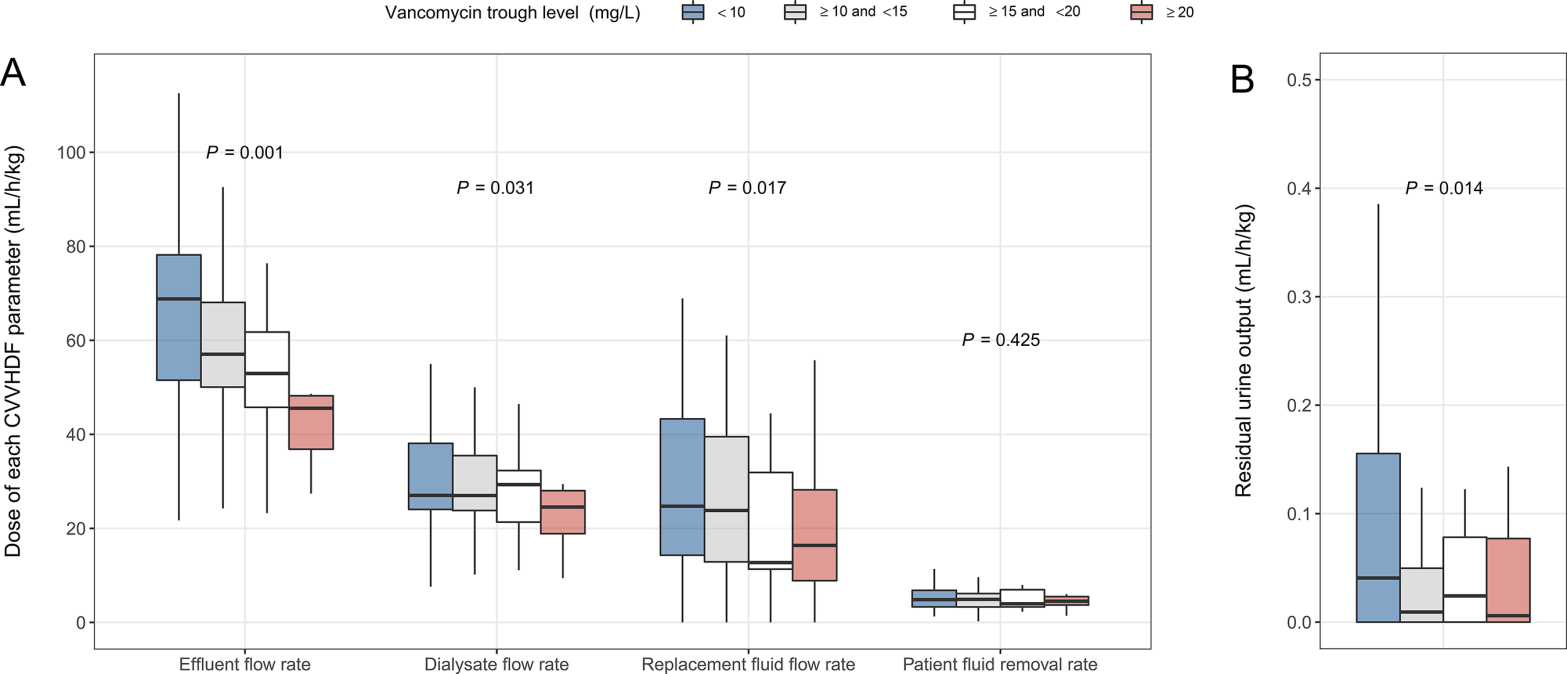
The dose of each CVVHDF parameter and residual urine output according to the vancomycin trough level groups. (A) The vancomycin trough levels were divided into 4 groups. The dose for each vancomycin trough level group was shown according to each CVVHDF parameter. (B) Residual diuresis showed a significant difference according to vancomycin trough level group. CVVHDF, continuous venovenous hemodiafiltration.

The samples of vancomycin TDM were divided into 4 groups (less than 10 mg/L, between 10–15 mg/L, between 15–20 mg/L, and 20 mg/L or more) according to their vancomycin trough levels. The doses of EFR (*P* = 0.001), DFR (*P* = 0.031), RFFR (*P* = 0.017), and residual urine output (*P* = 0.014) were significantly lower in the groups with a higher vancomycin trough level. On the contrary, PFRR showed no significant difference according to the groups (Table 3, Fig 3A and 3B).

**Table 3:** Relationship between vancomycin trough level group and each factor.

CVVHDF parameters	Vancomycin trough level groups				*P*
	< 10 mg/L (n = 47)	≥ 10 and < 15 mg/L (n = 49)	≥ 15 and < 20 mg/L (n = 15)	≥ 10 mg/L (n = 14)	
EFR (mL/h/kg)	68.82 (51.53–78.17)	57.05 (50.06–68.06)	52.95 (45.77–61.76)	45.56 (36.23–48.63)	0.001
DFR (mL/h/kg)	27.03 (24.04–38.09)	26.99 (23.81–35.48)	29.31 (21.34–32.3)	24.54 (18.04–28.46)	0.031
RFFR (mL/h/kg)	24.72 (14.3–43.29)	23.81 (12.89–39.5)	12.73 (11.33–31.9)	16.39 (8.12–29.66)	0.017
PFRR (mL/h/kg)	4.85 (3.31–6.82)	4.9 (3.29–6.14)	3.96 (3.3–6.97)	4.51 (3.69–5.7)	0.425
Residual urine output (mL/h/kg)	0.04 (0–0.16)	0.01 (0–0.05)	0.02 (0–0.08)	0.01 (0–0.08)	0.014

Data are presented as median (interquartile range).

CVVHDF, continuous venovenous hemodiafiltration; SE, standard error; EFR, effluent flow rate; DFR, dialysate flow rate; RFFR, replacement fluid flow rate; PFRR, patient fluid removal rate

## Discussion

This study was designed to analyze clinical and demographic parameters, to observe which parameters affect the serum concentration of vancomycin. The data analyzed from 177 pediatric samples of vancomycin TDM over a ten-year period demonstrated that DFR, RFFR, EFR, the proportion of RFFR, and residual urine output were significantly related to the serum concentrations of vancomycin.

In this study, the serum concentration of vancomycin decreased with increasing EFR, DFR, and RFFR. This is consistent with previous researches showing that vancomycin is excreted via hemofiltration and hemodialysis, and that the intensity of RFFR and DFR correlated with the clearance of vancomycin [5, 7, 13, 23–26]. It is also consistent with previous studies that show EFR to be a reliable predictor of antibiotic concentration in patients receiving CVVHDF [8, 27]. In the past, due to the use of low-flux hemofilter, drug clearance by CRRT, especially hemodialysis, was influenced by the size of drug molecule. However, in the recent years, high-flux hemofilters have replaced low-flux hemofilters, therefore having almost no influence on drug clearance according to the size of the drug molecule [3, 13]. Because all samples of vancomycin TDM included in this study received CVVHDF with a high-flux hemofilter AN69 [28, 29], it is possible that vancomycin could be effectively excreted by hemodialysis.

As the proportion RFFR increased, that is, the ratio of hemofiltration to hemodialysis increased, the serum concentration of vancomycin decreased significantly. This is in agreement with studies reporting that vancomycin is more effectively excreted by hemofiltration than by hemodialysis [16], but contrasts studies that recommend a higher vancomycin dose for patients receiving CVVHD or CVVHDF than CVVH [12, 15]. Because many factors related to the pharmacokinetics of vancomycin interact with each other in critically ill patients receiving CRRT [30], convection and diffusion may act in synergy with each other, causing an improved convection and over diffusion [11]. However, it is difficult to make a simple comparison with these studies, and in addition, the aforementioned studies were conducted on adults. It is known that the protein binding faction of vancomycin is significantly lower in children than in adults [18, 19]. Therefore, the possibility that vancomycin clearance by CVVHDF may be affected by the difference in the pharmacokinetic characteristics of vancomycin in these children compared with adults should be considered carefully.

In this study, to diminish the effect of residual renal function on vancomycin trough level, samples from patients with a urine output ≥ 0.5 mL/kg/h were excluded from the analyses. Nevertheless, even with negligible residual urine output of <0.5 mL/kg/hr, this study showed that vancomycin trough levels can be significantly affected, which is consistent with previous studies [31, 32]. More than a quarter of the samples were in the subtherapeutic range (<10mg/L [33, 34]) even if the administered median vancomycin dose in this patient group was 20.11 mg/kg/day; which is the recommended dose (20 mg/kg/day) for patients receiving CVVHDF. This phenomenon can be explained by vancomycin being excreted not only by CVVHDF, but also by residual diuresis. In fact, patients with a higher residual urine output had lower vancomycin trough levels, moreover, most of these patients were in the subtherapeutic group.

Although vancomycin clearance in patients receiving CVVHDF appears to be easily predicted by the known principles of hemofiltration, hemodialysis, and pharmacokinetic properties of vancomycin, it is recommended that TDM be performed instead of relying only on the predicted value because the actual clinically measured value is often different from the predicted value [3, 7, 8, 14, 35, 36]. In particular, vancomycin has a narrow therapeutic range, thus accurate prediction and maintenance of proper serum concentration are more important [20, 21]. For these reasons, the necessity of research based on actual clinically obtained results, as well as experimental research has emerged; and in fact, such clinical studies have been carried out [8, 30]. Nevertheless, only a few clinical researches include data on children. This study is significant in that there is no data comparing the proportion of hemofiltration and hemodialysis which affects the serum concentration of vancomycin in children receiving CVVHDF.

This study has several limitations. First, because this study was a retrospective study, controlling all the variables to exclude bias was difficult, and incomplete data restricted the extent of the study. An example being the duration of hemofilter use, which may have affected vancomycin clearance. However, we were unable to obtain this information. Second, this study derived a model through multiple regression analysis. If population pharmacokinetics were used, a more optimized model could be derived. Finally, this study was conducted at a PICU in a single center, and therefore caution is needed when applying the results of this study to other centers. Nevertheless, data obtained from this study can be the basis for future prospective studies. Also, it is important for bringing awareness to the necessity of studies on children receiving CVVHDF.

## Conclusions

In conclusion, this study demonstrated that serum concentration of vancomycin was associated with EFR, DFR, RFFR, the proportion of RFFR, and residual urine output in oliguric pediatric patients receiving CVVHDF. A well-designed multicenter study is needed to draw conclusions that are more specific and applicable at a broader level.

## Supporting information

S1 Table Raw data used in this study for statistical analyses can be found in S1 Table. (XLSX)

## References

[1] Bhargava D, Deshpande A, Sreekumar K, Koneru G, Rastogi S. Guidelines of the Infectious Diseases Society of America for the Treatment of Methicillin-Resistant Staphylococcus aureus Infections: As Applied to Oral and Maxillofacial Clinical Practice. Journal of maxillofacial and oral surgery. 2013;12(3):354–8. Epub 2014/01/17. doi: 10.1007/s12663-012-0374-6 ; PubMed Central PMCID: PMCPMC3777035. 24431869 10.1007/s12663-012-0374-6 PMC3777035

[2] Liu C, Bayer A, Cosgrove SE, Daum RS, Fridkin SK, Gorwitz RJ, et al Clinical practice guidelines by the infectious diseases society of america for the treatment of methicillin-resistant Staphylococcus aureus infections in adults and children: executive summary. Clinical infectious diseases: an official publication of the Infectious Diseases Society of America. 2011;52(3):285–92. Epub 2011/01/11. doi: 10.1093/cid/cir034 . 21217178 10.1093/cid/cir034

[3] Bugge JF. Pharmacokinetics and drug dosing adjustments during continuous venovenous hemofiltration or hemodiafiltration in critically ill patients. Acta anaesthesiologica Scandinavica. 2001;45(8):929–34. Epub 2001/09/29. . 11576041 10.1034/j.1399-6576.2001.450802.x

[4] Choi G, Gomersall CD, Tian Q, Joynt GM, Li AM, Lipman J. Principles of antibacterial dosing in continuous renal replacement therapy. Blood Purif. 2010;30(3):195–212. Epub 2010/10/07. doi: 10.1159/000321488 . 20924175 10.1159/000321488

[5] Frazee EN, Kuper PJ, Schramm GE, Larson SL, Kashani KB, Osmon DR, et al Effect of continuous venovenous hemofiltration dose on achievement of adequate vancomycin trough concentrations. Antimicrobial agents and chemotherapy. 2012;56(12):6181–5. Epub 2012/09/19. doi: 10.1128/AAC.00459-12 ; PubMed Central PMCID: PMCPmc3497205. 22985887 10.1128/AAC.00459-12 PMC3497205

[6] Kubin C, Dzierba A. The effects of continuous renal replacement on anti-infective therapy in the critically ill. Journal of Pharmacy Practice. 2005;18(2):109–17.

[7] Wilson FP, Berns JS. Vancomycin levels are frequently subtherapeutic during continuous venovenous hemodialysis (CVVHD). Clinical nephrology. 2012;77(4):329–31. Epub 2012/03/27. doi: 10.5414/CN106993 ; PubMed Central PMCID: PMCPmc3359699. 22445477 10.5414/CN106993 PMC3359699

[8] Roberts DM, Liu X, Roberts JA, Nair P, Cole L, Roberts MS, et al A multicenter study on the effect of continuous hemodiafiltration intensity on antibiotic pharmacokinetics. Critical care (London, England). 2015;19:84 Epub 2015/04/18. doi: 10.1186/s13054-015-0818-8 ; PubMed Central PMCID: PMCPmc4404619. 25881576 10.1186/s13054-015-0818-8 PMC4404619

[9] Eiland LS, English TM, Eiland EH, 3rd. Assessment of vancomycin dosing and subsequent serum concentrations in pediatric patients. The Annals of pharmacotherapy. 2011;45(5):582–9. Epub 2011/04/28. doi: 10.1345/aph.1P588 . 21521865 10.1345/aph.1P588

[10] Boereboom FT, Ververs FF, Blankestijn PJ, Savelkoul TJ, van Dijk A. Vancomycin clearance during continuous venovenous haemofiltration in critically ill patients. Intensive care medicine. 1999;25(10):1100–4. Epub 1999/11/07. . 10551965 10.1007/s001340051018

[11] Brunet S, Leblanc M, Geadah D, Parent D, Courteau S, Cardinal J. Diffusive and convective solute clearances during continuous renal replacement therapy at various dialysate and ultrafiltration flow rates. American journal of kidney diseases: the official journal of the National Kidney Foundation. 1999;34(3):486–92. Epub 1999/09/02. doi: 10.1053/ajkd03400486 . 10469859 10.1016/s0272-6386(99)70076-4

[12] Heintz BH, Matzke GR, Dager WE. Antimicrobial dosing concepts and recommendations for critically ill adult patients receiving continuous renal replacement therapy or intermittent hemodialysis. Pharmacotherapy. 2009;29(5):562–77. Epub 2009/04/29. doi: 10.1592/phco.29.5.562 . 19397464 10.1592/phco.29.5.562

[13] Joy MS, Matzke GR, Frye RF, Palevsky PM. Determinants of vancomycin clearance by continuous venovenous hemofiltration and continuous venovenous hemodialysis. American journal of kidney diseases: the official journal of the National Kidney Foundation. 1998;31(6):1019–27. Epub 1998/06/19. . 9631848 10.1053/ajkd.1998.v31.pm9631848

[14] Lewis SJ, Mueller BA. Antibiotic dosing in critically ill patients receiving CRRT: underdosing is overprevalent. Semin Dial. 2014;27(5):441–5. Epub 2014/09/11. doi: 10.1111/sdi.12203 . 25204875 10.1111/sdi.12203

[15] Trotman RL, Williamson JC, Shoemaker DM, Salzer WL. Antibiotic dosing in critically ill adult patients receiving continuous renal replacement therapy. Clinical infectious diseases: an official publication of the Infectious Diseases Society of America. 2005;41(8):1159–66. Epub 2005/09/16. doi: 10.1086/444500 . 16163635 10.1086/444500

[16] Jeffrey RF, Khan AA, Prabhu P, Todd N, Goutcher E, Will EJ, et al A comparison of molecular clearance rates during continuous hemofiltration and hemodialysis with a novel volumetric continuous renal replacement system. Artificial organs. 1994;18(6):425–8. Epub 1994/06/01. . 8060251 10.1111/j.1525-1594.1994.tb02228.x

[17] De Bock V, Verbeelen D, Maes V, Sennesael J. Pharmacokinetics of vancomycin in patients undergoing haemodialysis and haemofiltration. Nephrology, dialysis, transplantation: official publication of the European Dialysis and Transplant Association—European Renal Association. 1989;4(7):635–9. Epub 1989/01/01. . 2510061

[18] De Cock PA, Desmet S, De Jaeger A, Biarent D, Dhont E, Herck I, et al Impact of vancomycin protein binding on target attainment in critically ill children: back to the drawing board? The Journal of antimicrobial chemotherapy. 2017;72(3):801–4. Epub 2016/12/22. doi: 10.1093/jac/dkw495 . 27999035 10.1093/jac/dkw495

[19] Oyaert M, Spriet I, Allegaert K, Smits A, Vanstraelen K, Peersman N, et al Factors impacting unbound vancomycin concentrations in different patient populations. Antimicrobial agents and chemotherapy. 2015;59(11):7073–9. Epub 2015/09/10. doi: 10.1128/AAC.01185-15 ; PubMed Central PMCID: PMCPMC4604401. 26349820 10.1128/AAC.01185-15 PMC4604401

[20] Rybak M, Lomaestro B, Rotschafer JC, Moellering R Jr., Craig W, Billeter M, et al Therapeutic monitoring of vancomycin in adult patients: a consensus review of the American Society of Health-System Pharmacists, the Infectious Diseases Society of America, and the Society of Infectious Diseases Pharmacists. American journal of health-system pharmacy: AJHP: official journal of the American Society of Health-System Pharmacists. 2009;66(1):82–98. Epub 2008/12/25. doi: 10.2146/ajhp080434 . 19106348 10.2146/ajhp080434

[21] Matsumoto K, Takesue Y, Ohmagari N, Mochizuki T, Mikamo H, Seki M, et al Practice guidelines for therapeutic drug monitoring of vancomycin: a consensus review of the Japanese Society of Chemotherapy and the Japanese Society of Therapeutic Drug Monitoring. Journal of infection and chemotherapy: official journal of the Japan Society of Chemotherapy. 2013;19(3):365–80. Epub 2013/05/16. doi: 10.1007/s10156-013-0599-4 . 23673472 10.1007/s10156-013-0599-4

[22] Kliegman RM, Stanton BF, Schor NF, Geme JW St. III, editors. Nelson textbook of pediatrics. 20th ed. Philadelphia, PA: Elsevier/Saunders; 2015.

[23] Paciullo CA, Harned KC, Davis GA, Connor MJ Jr., Winstead PS. Vancomycin clearance in high-volume venovenous hemofiltration. The Annals of pharmacotherapy. 2013;47(3):e14 Epub 2013/02/14. doi: 10.1345/aph.1Q488 . 23404801 10.1345/aph.1Q488

[24] Park I, Lee SA, Lim SK, Yu S, Jang EJ, Moon EJ, et al Vancomycin Pharmacokinetics in Oliguric Patients Undergoing Continuous Venovenous Hemodialysis and Continuous Venovenous Hemodiafiltration. Korean Journal of Nephrology. 2010;29(5):585–92.

[25] Petejova N, Martinek A, Zahalkova J, Duricova J, Brozmannova H, Urbanek K, et al Vancomycin pharmacokinetics during high-volume continuous venovenous hemofiltration in critically ill septic patients. Biomedical papers of the Medical Faculty of the University Palacky, Olomouc, Czechoslovakia. 2014;158(1):65–72. Epub 2012/11/08. doi: 10.5507/bp.2012.092 . 23132513 10.5507/bp.2012.092

[26] Troyanov S, Cardinal J, Geadah D, Parent D, Courteau S, Caron S, et al Solute clearances during continuous venovenous haemofiltration at various ultrafiltration flow rates using Multiflow-100 and HF1000 filters. Nephrology, dialysis, transplantation: official publication of the European Dialysis and Transplant Association—European Renal Association. 2003;18(5):961–6. Epub 2003/04/11. . 12686672 10.1093/ndt/gfg055

[27] Jamal JA, Udy AA, Lipman J, Roberts JA. The impact of variation in renal replacement therapy settings on piperacillin, meropenem, and vancomycin drug clearance in the critically ill: an analysis of published literature and dosing regimens*. Critical care medicine. 2014;42(7):1640–50. Epub 2014/03/29. doi: 10.1097/CCM.0000000000000317 . 24674926 10.1097/CCM.0000000000000317

[28] Collins DM, Lambert MB, Tannenbaum JS, Oliverio M, Schwab SJ. Tolerance of hemodialysis: a randomized prospective trial of high-flux versus conventional high-efficiency hemodialysis. J Am Soc Nephrol. 1993;4(2):148–54. Epub 1993/08/01. . 8400077 10.1681/ASN.V42148

[29] Thomas M, Moriyama K, Ledebo I. AN69: Evolution of the world's first high permeability membrane. Contributions to nephrology. 2011;173:119–29. Epub 2011/08/26. doi: 10.1159/000328961 . 21865784 10.1159/000328961

[30] Udy AA, Covajes C, Taccone FS, Jacobs F, Vincent JL, Lipman J, et al Can population pharmacokinetic modelling guide vancomycin dosing during continuous renal replacement therapy in critically ill patients? International journal of antimicrobial agents. 2013;41(6):564–8. Epub 2013/03/12. doi: 10.1016/j.ijantimicag.2013.01.018 . 23473944 10.1016/j.ijantimicag.2013.01.018

[31] Ulldemolins M, Soy D, Llaurado-Serra M, Vaquer S, Castro P, Rodriguez AH, et al Meropenem population pharmacokinetics in critically ill patients with septic shock and continuous renal replacement therapy: influence of residual diuresis on dose requirements. Antimicrobial agents and chemotherapy. 2015;59(9):5520–8. Epub 2015/07/01. doi: 10.1128/AAC.00712-15 ; PubMed Central PMCID: PMCPMC4538468. 26124172 10.1128/AAC.00712-15 PMC4538468

[32] Ulldemolins M, Vaquer S, Llaurado-Serra M, Pontes C, Calvo G, Soy D, et al Beta-lactam dosing in critically ill patients with septic shock and continuous renal replacement therapy. Critical care (London, England). 2014;18(3):227 Epub 2014/07/22. doi: 10.1186/cc13938 ; PubMed Central PMCID: PMCPMC4075152. 25042938 10.1186/cc13938 PMC4075152

[33] Sakoulas G, Eliopoulos GM, Moellering RC Jr., Novick RP, Venkataraman L, Wennersten C, et al Staphylococcus aureus accessory gene regulator (agr) group II: is there a relationship to the development of intermediate-level glycopeptide resistance? The Journal of infectious diseases. 2003;187(6):929–38. Epub 2003/03/28. doi: 10.1086/368128 . 12660939 10.1086/368128

[34] Tsuji BT, Rybak MJ, Lau KL, Sakoulas G. Evaluation of accessory gene regulator (agr) group and function in the proclivity towards vancomycin intermediate resistance in Staphylococcus aureus. Antimicrobial agents and chemotherapy. 2007;51(3):1089–91. Epub 2006/12/13. doi: 10.1128/AAC.00671-06 ; PubMed Central PMCID: PMCPMC1803123. 17158941 10.1128/AAC.00671-06 PMC1803123

[35] Bouman CS, van Kan HJ, Koopmans RP, Korevaar JC, Schultz MJ, Vroom MB. Discrepancies between observed and predicted continuous venovenous hemofiltration removal of antimicrobial agents in critically ill patients and the effects on dosing. Intensive care medicine. 2006;32(12):2013–9. Epub 2006/10/18. doi: 10.1007/s00134-006-0397-x . 17043848 10.1007/s00134-006-0397-x

[36] Omrani AS, Mously A, Cabaluna MP, Kawas J, Albarrak MM, Alfahad WA. Vancomycin therapy in critically ill patients on continuous renal replacement therapy; are we doing enough? Saudi pharmaceutical journal: SPJ: the official publication of the Saudi Pharmaceutical Society. 2015;23(3):327–9. Epub 2015/06/25. doi: 10.1016/j.jsps.2014.08.005 ; PubMed Central PMCID: PMCPmc4475842. 26106281 10.1016/j.jsps.2014.08.005 PMC4475842

